# Mechanisms of 8‐aminoquinoline induced haemolytic toxicity in a G6PDd humanized mouse model

**DOI:** 10.1111/jcmm.17362

**Published:** 2022-06-03

**Authors:** Siobhan Flaherty, Pamela Strauch, Mahdi Maktabi, Brandon S. Pybus, Gregory Reichard, Larry A Walker, Rosemary Rochford

**Affiliations:** ^1^ Department of Immunology and Microbiology The University of Colorado School of Medicine Aurora Colorado USA; ^2^ 8394 Division of Experimental Therapeutics Walter Reed Army Institute of Research Silver Spring Maryland USA; ^3^ 8394 Department of Drug Discovery Experimental Therapeutics Branch Walter Reed Army Institute of Research Silver Spring Maryland USA; ^4^ National Center for Natural Products Research and Department of Biomolecular Sciences School of Pharmacy The University of Mississippi University Mississippi USA

**Keywords:** 8‐aminoquinoline, eryptosis, extravascular haemolysis, glucose‐6‐phosphate dehydrogenase deficiency, haemolytic anaemia, haemolytic toxicity, intravascular haemolysis, primaquine, stress erythropoiesis, tafenoquine

## Abstract

Primaquine (PQ) and Tafenoquine (TQ) are clinically important 8‐aminoquinolines (8‐AQ) used for radical cure treatment of *P*. *vivax* infection, known to target hepatic hypnozoites. 8‐AQs can trigger haemolytic anaemia in individuals with glucose‐6‐phosphate dehydrogenase deficiency (G6PDd), yet the mechanisms of haemolytic toxicity remain unknown. To address this issue, we used a humanized mouse model known to predict haemolytic toxicity responses in G6PDd human red blood cells (huRBCs). To evaluate the markers of eryptosis, huRBCs were isolated from mice 24–48 h post‐treatment and analysed for effects on phosphatidylserine (PS), intracellular reactive oxygen species (ROS) and autofluorescence. Urinalysis was performed to evaluate the occurrence of intravascular and extravascular haemolysis. Spleen and liver tissue harvested at 24 h and 5–7 days post‐treatment were stained for the presence of CD169+ macrophages, F4/80+ macrophages, Ter119+ mouse RBCs, glycophorin A+ huRBCs and murine reticulocytes (muRetics). G6PDd‐huRBCs from PQ/TQ treated mice showed increased markers for eryptosis as early as 24 h post‐treatment. This coincided with an early rise in levels of muRetics. Urinalysis revealed concurrent intravascular and extravascular haemolysis in response to PQ/TQ. Splenic CD169+ macrophages, present in all groups at day 1 post‐dosing were eliminated by days 5–7 in PQ/TQ treated mice only, while liver F4/80 macrophages and iron deposits increased. Collectively, our data suggest 8‐AQ treated G6PDd‐huRBCs have early physiological responses to treatment, including increased markers for eryptosis indicative of oxidative stress, resulting in extramedullary haematopoiesis and loss of splenic CD169+ macrophages, prompting the liver to act as the primary site of clearance.

## 1 INTRODUCTION

Glucose‐6‐phosphate dehydrogenase deficiency (G6PDd) affects up to 500 million people worldwide and is considered the most common human hemoglobinopathy.^1^, ^2^, ^3^ Typically asymptomatic, a key clinical feature of G6PDd is haemolytic anaemia, which results from infection, exposure to certain medications and the consumption of fava beans.^1^, ^4^ Carriers of G6PDd variants are found predominantly in Africa, the Mediterranean, Middle East and Southeast Asia, with the highest allelic frequencies reported at up to ~30% in regions of sub‐Saharan Africa and the Arabian Peninsula.^5^ The prevalence of G6PDd and its geographic overlap with areas of malaria endemicity suggests that this enzymopathy confers protection against malaria mortality.^6^, ^7^, ^8^ However, the pervasiveness of G6PDd in these regions presents an obstacle for global malarial elimination efforts.

Five species of *Plasmodium* are known to cause malaria infection in humans: *P*. *falciparum*, *P*. *vivax*, *P*. *ovale*, *P*. *malariae* and *P*. *knowlesi*. Of these, *P*. *falciparum* and *P*. *vivax* are linked to the majority of infections worldwide with an estimated 229 million cases occurring in 2019.^9^ A unique feature of *P*. *vivax* and *P*. *ovale* infection is the liver‐stage component, where parasites can remain dormant as hypnozoites, relapsing with unpredictability, potentially years after infection.^10^, ^11^ Therefore, malaria elimination campaigns will need to target both active as well as latent hypnozoite infections.^12^

Primaquine (PQ) and the more recently approved drug, tafenoquine (TQ) belong to the class of compounds known as 8‐aminoquinolines (8‐AQ). They are the only licensed anti‐malarial drugs which act by targeting the hepatic hypnozoite forms of *Plasmodium* and thus are used to cure relapsing malaria. However, this class of compounds can cause non‐immune haemolytic anaemia in G6PDd individuals preventing widespread application for the radical cure of *P*. *vivax*.^12^ Studies dating back to the 1950’s showed the kinetics of haemolysis following treatment with PQ.^13^, ^14^ TQ‐associated RBC haemolysis has more recently been reported in a study which monitored effects of TQ treatment in heterozygous G6PDd females.^15^ As yet, there has not been a direct comparison of TQ to PQ haemolytic responses or a viable method for mitigating haemolytic anaemia in G6PDd individuals.

There are two commonly recognized modes of RBC loss that occur during haemolytic anaemia: intravascular or extravascular haemolysis.^16^ Intravascular haemolysis—considered more severe—is defined as lysis of RBCs, whereby the contents of the cells are released into circulation. During extravascular haemolysis, RBCs are removed by the mononuclear phagocytic system in the liver and spleen—the normal method of removal of senescent RBCs.^17^ RBCs exposed to oxidative stress can display markers of programmed cell death referred to as ‘eryptosis’, which leads to extravascular removal, and is the correlate of apoptosis in nucleated cells..^18^, ^19^, ^20^ Markers of eryptosis include increased cytosolic calcium levels, externalized phosphatidylserine (PS) expression, membrane changes and increased autofluorescence—a marker linked to the presence of Heinz bodies.^21^, ^22^, ^23^ Extravascular clearance of eryptotic erythrocytes protect against the broad release of toxic RBC contents, particularly haemoglobin.

During homeostasis, bone marrow is the site of erythropoiesis and is balanced by removal of ageing or damaged RBCs through splenic and hepatic macrophages.^22^ However, during anaemic stress and inflammation, to account for the rising demand for new erythrocytes, extramedullary production of splenic erythroid progenitors compensate for RBC loss.^24^, ^25^ Stress erythropoiesis has been studied in animal models; however, it is unknown if this occurs clinically during haemolytic anaemia.^26^, ^27^

Due to the metabolic activation requirement of 8‐AQs, in vitro models are inadequate for understanding the mechanisms of haemolytic toxicity in G6PDd RBCs.^28^ We previously developed the G6PDd human RBC (huRBC)‐NOD/SCID humanized mouse model (herein referred to as G6PDd‐huRBC engrafted mice).^29^ This model has been a validated screening tool for the haemolytic potential of anti‐malarial drugs in preclinical development, where drug‐induced haemolytic toxicity is indicated by loss of huRBCs, elevation of murine reticulocytes (muRetic), decline in haematocrit and splenomegaly.^29^ In this study, the G6PDd‐huRBC engrafted humanized mouse model was utilized to understand the time course and identify the underlying mechanisms of these responses to 8‐AQ treatment using PQ and TQ.

## 2 METHODS

### 2.1 Mice

NOD.CB17‐Prkdc^scid^/J mice (NOD/SCID) were purchased from the Jackson Laboratories (Bar Harbor, ME). Mice ranged in age from 8–10 weeks and were maintained in a pathogen–free facility at the CU Anschutz Medical Center vivarium. Research was conducted under an approved animal use protocol in an AAALAC International‐accredited facility in compliance with the Animal Welfare Act and all other federal statutes and regulations relating to animals and experiments involving animals, and adheres to principles stated in the Guide for Care and Use of Laboratory Animals, NRC Publication, 2011 edition.

### 2.2 HuRBC collection and processing

G6PD‐deficient volunteer blood donors were recruited through the Walter Reed Army Institute of Research (WRAIR), (Silver Spring, MD), under the approved IRB protocol 2567.04. Donor G6PD levels for A–genotype used in these experiments ranged from 1.9–2.9 U/g HgB. Blood was collected and processed as previously described.^29^

### 2.3 HuRBC engraftment

NOD/SCID mice received intraperitoneal injections of 5 × 10^9^ G6PDd‐huRBCs daily for 14 consecutive days as previously described.^29^ Post‐engraftment, tail‐vein blood was collected for flow cytometry and stained for mouse red blood cells (muRBC) with anti‐TER‐119 (clone TER‐119; BD Pharmingen, Franklin Lakes), huRBC with anti‐Glycophorin A (clone YTH89.1; Abcam, Cambridge, MA), and muRetics with anti‐CD71 (clone R172717; Invitrogen, Waltham, MA). Inclusion criteria for all experiments was >60% circulating peripheral huRBCs. Mice were subsequently randomized into the experimental groups.

### 2.4 Compound preparation and administration

PQ and TQ were provided by WRAIR, freshly reconstituted in PBS (GE Lifesciences) and HEC‐Tween (0.5% HEC, 0.1%, Tween80), respectively, and protected from light. Vehicle control (VC) groups received PBS only. Drug doses are adjusted for molecular weight of the salt, and are expressed in terms of the base.

### 2.5 Flow cytometric analysis of G6PDd‐huRBC

Flow cytometry was employed to assess changes in PS, reactive oxygen species (ROS) and autofluorescence, using blood collected at 24‐ and 48‐ h post‐dosing. Mice received one dose (p.o.) of the following: VC, PQ 15 mpk or TQ 10 mpk. Blood (10 µl) was collected via tail vein puncture and placed in 100 µl of PBS containing lithium‐heparin. RBCs were stained with anti‐Glycophorin A, with all steps performed at room temperature (RT) and protected from light.

Eryptosis was examined using Annexin‐V and propidium iodine (PI) staining, using the Annexin‐V‐FITC Apoptosis Detection Kit (Abcam), according to the manufacturer's protocol and as previously described.^30^ ROS levels were determined by staining with 2’,7’‐dichlorodihydrofluorescein‐diacetate (DCFDA) (Sigma, Saint Louis, MO) in Ringer solution (Boston BioProducts, Ashland, MA) as previously described.^30^ Autofluorescence was measured by the methods previously described.^31^

All flow cytometry was performed using a Cytek^®^ Northern Lights flow cytometer, 10^5^ events were collected and results were analysed using FlowJo™ Software (BD) with graphs generated using GraphPad Prism 8.0 software (GraphPad Software, Inc., San Diego, CA). Sample gating strategy for Annexin‐PI and autofluorescence is provided in Figure S1.

### 2.6 Heinz body staining

G6PDd‐huRBC mice were administered one dose (p.o.) of VC, PQ 15 mpk or TQ 10 mpk. Blood was collected at 24 (VC, PQ) or 48 h (TQ) post‐dosing and stained with a. 5% crystal violet solution (Thermo Fisher, Waltham, MA), as previously described.^32^ Images were acquired using a Leica DM750 microscope and Leica Acquire software 10.10 (Leica Camera AG, Wetzlar, Germany) at 100x with oil immersion. Images were processed using ImageJ software and Adobe Photoshop Creative Cloud (Adobe Systems, San Jose, CA).^33^

### 2.7 Urine collection and analysis

G6PDd‐huRBC mice were grouped and treated with either VC × 3, PQ 15 mpk/d × 3 and TQ 5 mpk × 1. At 50 h post‐initial doses urine was collected and stored at 4°C prior to analysis. Urinalysis was performed by the CU CPSR Core laboratory and included testing for protein, glucose, occult blood, bilirubin, and urobilinogen using Fisherbrand 10SG Urine Reagent Strips. Urine pH was confirmed via MColorpHast™ pH‐indicator strips (non‐bleeding) (MilliporeSigma, Darmstadt, Germany).

### 2.8 Immunofluorescence and immunohistochemistry

Liver and spleen tissue were collected at 24 h (early) and 5–7 days (late) post‐compound administration for immunofluorescent (IF) staining. The early 24 h time point tissue was harvested from mice which received 1 dose (p.o) of VC, PQ 15 mpk or TQ 10 mpk. At the late 5–7 days timepoint mice received either VC × 3, PQ 15 mpk/d × 3 or TQ 10 mpk × 1. Engrafted and non‐engrafted spleen tissue and engrafted, non‐engrafted and wild type C57‐BL/6J mouse hepatic tissue was stained for comparison (Figures S2A,B). Spleen and liver tissue were embedded in Tissue‐Tek OCT (Sakura Finetek, Torrance, CA), snap frozen and stored at −80°C, sectioned using a cryostat (CryoJane adaptation, Leica) into 6 μm thick sections and transferred onto glass slides. Slides were dried for 30 min, fixed for 30 sec in 50% acetone, then 3 min in 100% acetone. Sections were rehydrated in PBS for 20 min, blocked with rat serum including rat anti‐mouse CD16/CD32 2.4G2, Fc block (1:100) (BD), in a 2% BSA/.05% Tween20/PBS solution for 20 min. Blocking solution was removed with a fluorescent‐labelled antibody cocktail added for 45 min. Slides were washed 3x with PBS, followed by the addition of Vectamount Mounting Media (Vector Laboratories, Burlingame, CA), and stored at RT for up to 24 h before image capture. The following antibodies were used: Glycophorin A CD235a (clone YTH89.1; Invitrogen), TER‐119 (clone Ter‐119; BD), CD169 (clone 3D6.112; Biolegend, San Diego, CA), CD71 (clone R17217; Invitrogen) and F4/80 (clone BM8; Biolegend). Sections were captured on an Eclipse TE 2000 microscope (Nikon, Tokyo, Japan) and analyzed using Slidebook 6.0 software (Intelligent Imaging Innovations). Fluorochrome settings were set to equivalent intensities among all samples for analysis, by utilizing single stained isotype controls, with all files exported using equivalent settings and file formats.

Prussian Blue staining method for detection of ferric iron was performed on Day 7 liver tissue from mice dosed (p.o.) with VC × 3, PQ 15 mpk/d × 3 or TQ 10 mpk × 1. Tissue was embedded in Tissue‐Tek OCT (Sakura Finetek), snap frozen, stored at −80°C, then sectioned using a cryostat and stained using an Iron Stain Kit (Abcam) according to the manufacture's protocol. Images were acquired and processed as described above in the Heinz body staining section, at 40x magnification.

### 2.9 Statistical analysis

Statistical significance was evaluated using GraphPad PRISM 8.0 software (GraphPad Software, Inc.). Differences between VC, PQ and TQ groups were evaluated using an ordinary one‐way analysis of variance (ANOVA) followed by Dunnett's multiple comparison test for cytometric flow analysis of huRBCs, muRetics and spleen weight. For analysis of Annexin‐V binding, DCFDA and autofluorescence an ordinary two‐way ANOVA was performed followed by Tukey's multiple comparisons test. Significance was noted as follows: *p < .05; **p < .01; ***p < .001; ****p < .0001. Error bars denote the standard error of the mean.

## 3 RESULTS

### 3.1 8‐AQ induced haemolytic toxicity in NOD/SCID mice engrafted with G6PDd‐huRBCs

**FIGURE 1:**
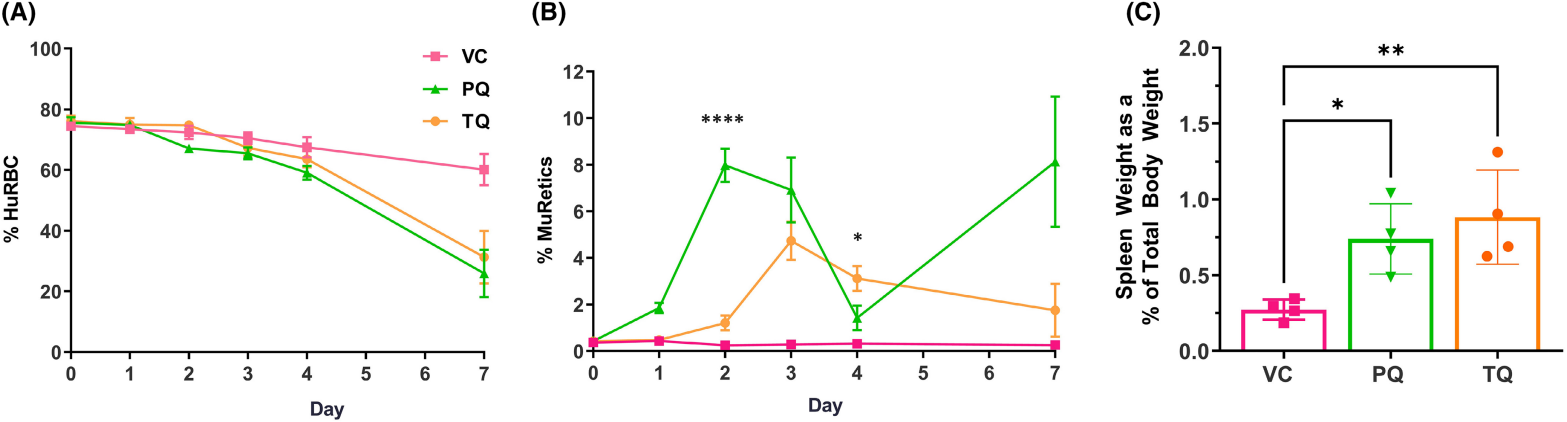
In response to 8‐AQ treatment G6PDd‐huRBCs decline and muRetics increase, resulting in enlarged spleen tissue. Mice were engrafted with G6PDd‐huRBCs and treated with VC × 1, PQ 15 mpk × 1 or TQ 10 mpk × 1 and monitored for changes to huRBCs and muRetics, with terminal spleen weight measured at 7 days post‐8‐AQ treatment. Results are for 4 mice per group. The kinetics of (A) huRBC (Glycophorin‐A+, FITC) and (B) muRetic (TER‐119+, PE) levels are shown. (C) Spleen weight was also assessed at the termination of experiment and are presented as normalized data relative to total body weight. Data are the representative of 3 independent experiments

We have previously shown that in G6PDd‐huRBC engrafted mice, PQ induces both the loss of huRBCs and increase of muRetics resulting in elevated spleen weights.^29^ TQ has been shown to have haemolytic potential in humans with G6PDd but has not been extensively analysed for haemolytic toxicity. To analyse the effects of TQ and PQ on haemolytic toxicity G6PDd‐huRBC mice received one dose of VC, PQ (15 mpk) or TQ (10 mpk). Flow cytometry performed on days 1–4, and 7 post‐treatment, monitored changes in huRBC and muRetic populations in response to treatment (Figure 1A,B). Analysis showed huRBC levels remained high in the VC group over the course of 7 days while the PQ and TQ treated groups exhibited statistically significant decreases in huRBC by Day 7 compared with VC (*p* = .017, *p* = .039, respectively). MuRetic analysis showed that VC treated mice had stable reticulocyte levels remaining under 1% throughout the experiment. In contrast, PQ treated mice exhibited a bimodal pattern of muRetic increase, and in TQ treated mice, no increase in muRetics was observed until Day 2–3, which then declined until Day 7. Spleen weight was collected on Day 7 and calculated as a percent of body weight. huRBC decline resulted in increased spleen weight (Figure 1C), in both PQ (*p* = .031) and TQ (*p* = .008) treated groups, consistent with a model of induced extramedullary erythropoiesis. These results indicate both TQ and PQ treatment resulted in haemolytic toxicity in G6PDd‐huRBC mice.

### 3.2 Early indications of oxidative stress and eryptosis in G6PDd‐huRBCs in response to 8‐AQ treatment

**FIGURE 2:**
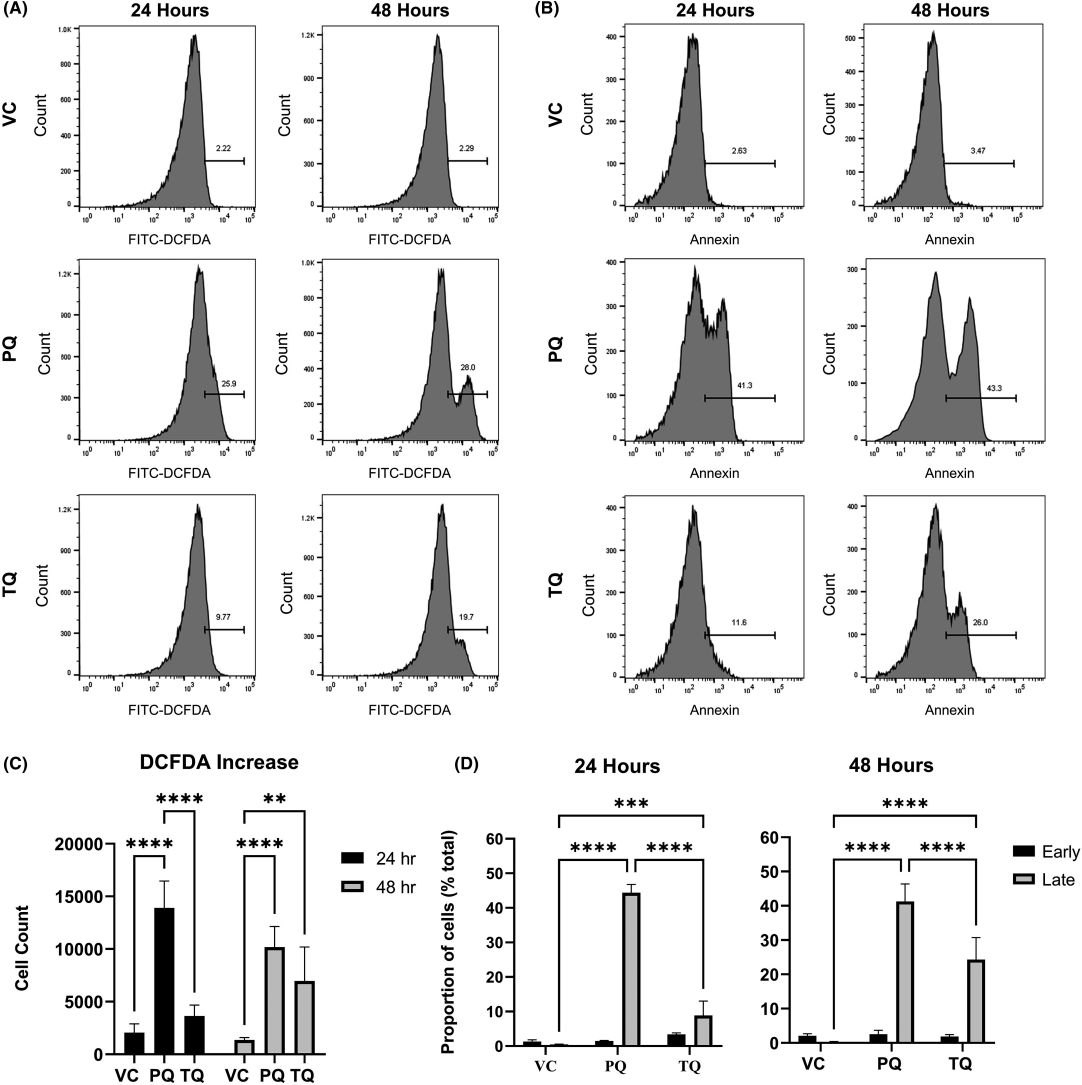
ROS and PS increase in response to 8‐AQ treatment. Mice were engrafted with G6PDd‐huRBCs and treated with VC × 1, PQ 15 mpk × 1 or TQ 10 mpk × 1 with flow cytometry performed to measure effects of treatment on ROS and PS levels in huRBCs at 24‐ and 48‐ h post‐treatment. huRBCs were stained with DCFDA (FITC) to measure ROS production (A) and Annexin‐V binding (FITC) (B). Significant differences in ROS production (C) and PS (D) were reported at 24 and 48 h. PS (D) showed significant changes in late (end‐stage) apoptotic cell populations (late), with minimal changes in early apoptotic events (early). Results are for 4 mice per group and data are representative of 3 independent experiments. *p < .05; **p < .01; ***p < .001; ****p < .0001; ANOVA with the Dunnett's multiple comparisons test. Error bars denote the standard error of the mean

The early increase in muRetics in response to PQ and TQ treatment led us to evaluate markers of oxidative stress and eryptosis at early timepoints post‐treatment. The following three markers were evaluated by flow cytometry: PS, intracellular ROS and autofluorescence. Increases in PS on the surface of RBCs are an indicator of membrane damage, intracellular ROS provides information on oxidative stress within the RBC and elevation of autofluorescence has been linked to Heinz body formation and clearance.^31^ G6PDd‐huRBC engrafted mice received one dose of VC, PQ (15 mpk) or TQ (10 mpk). RBCs were analysed at 24‐ and 48‐ h post‐dosing. At both 24 h and 48 h post‐dosing, increased levels of DCFDA were observed in the PQ treated group compared with VC (*p* = < .0001). In contrast, TQ treated mice did not show a significant increase in DCFDA compared to VC until 48 h post‐dosing (*p* = .008) (Figure 2A,C). Significant differences were found between DCFDA levels in PQ and TQ treated huRBCs in mice at 24 h.

Evaluation of PS on the huRBC cell surface followed a similar pattern with a very high percent of Annexin+cells in the PQ treated mice compared to VC at 24 and 48 h. Annexin+cells in TQ treated mice were lower compared with PQ treated mice at 24 h and increased by 48 h. Differences in Annexin were statistically significant between PQ and VC compared at 24 and 48 h (*p* = <.0001), and between TQ and VC at 24 (*p* = .0001) and 48 h (*p* = <.0001). Comparisons of PQ to TQ Annexin binding levels were significant at both 24 and 48 h (*p* = <.0001) (Figure 2B,D).

**FIGURE 3:**
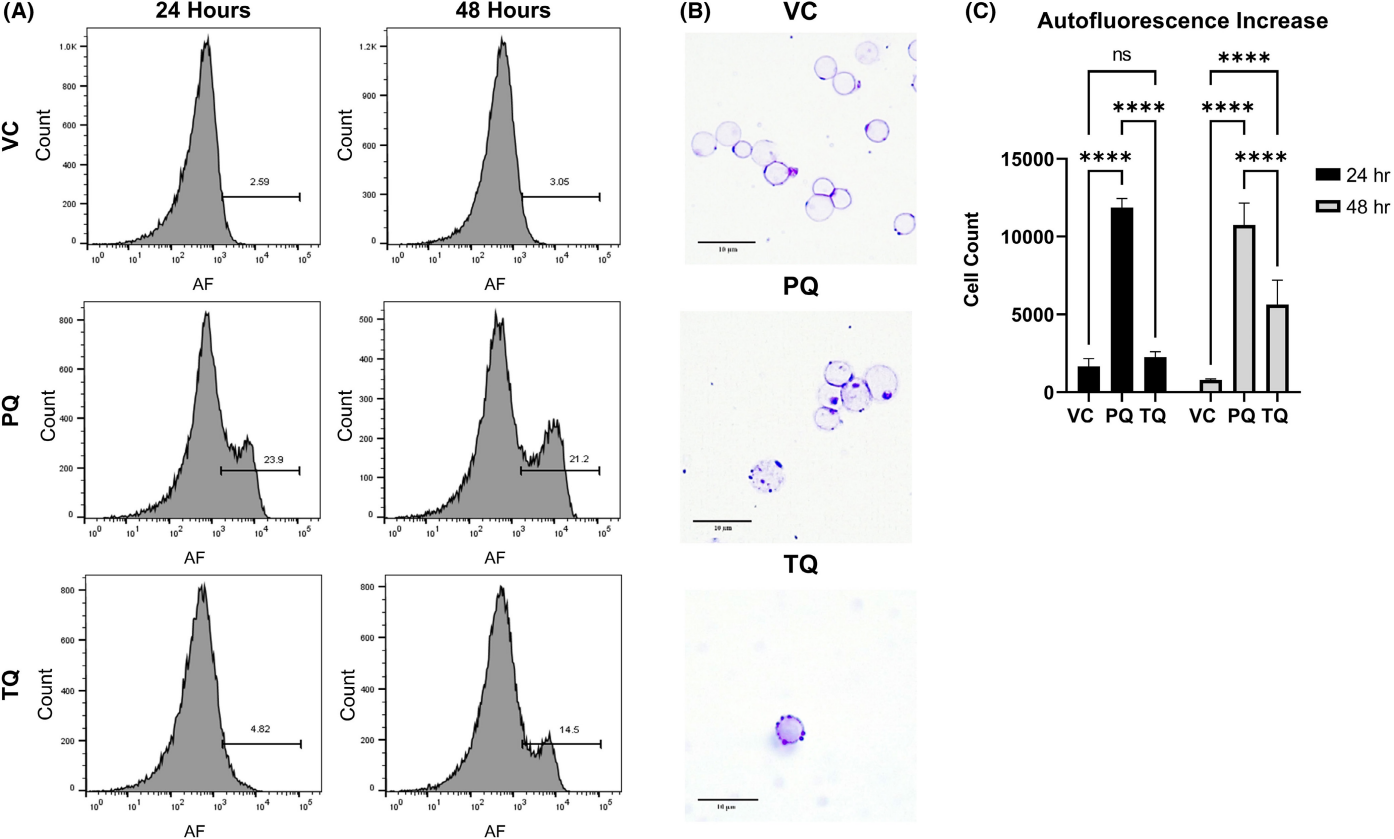
G6PDd‐huRBCs have autofluorescence increases with evidence for Heinz body formation at 24–48 h post 8‐AQ treatment. Mice were engrafted with G6PDd‐huRBCs and treated with VC × 1, PQ 15 mpk × 1 or TQ 10 mpk × 1 with flow cytometry performed to measure the effects of treatment on huRBC autofluorescence at 24 and 48 h post‐treatment. Results are for 4 mice per group and representative of 3 independent experiments. Autofluorescence increases above levels documented in G6PDd untreated cells was used as a marker to define effect on huRBCs (A). Significant differences in autofluorescence (C) was reported at 24 and 48 h. Crystal Violet staining (B) was performed on whole blood at 24 h for VC, 24 h for PQ and 48 h for TQ, with representative images chosen. Gating strategy for autofluorescence measurements are provided in Supplemental Figure 1. Data are representative of 3 independent experiments. *p < .05; **p < .01; ***p < .001; ****p < .0001; ANOVA with the Dunnett's multiple comparisons test. Error bars denote the standard error of the mean. Crystal violet images were captured at 100x magnification using a Leica DM750 microscope and Leica Acquire software 10.10. Images were further processes using ImageJ software^33^ to add scale bars and Adobe Photoshop Creative Cloud for image cropping

Elevated autofluorescence was measured at 24‐ and 48‐ h post‐dosing in huRBCs. Significant differences were found with PQ treatment at 24 h (*p* = <.0001) and at 48 h (*p* = <.0001) compared to VC treated mice (Figure 3A,C). TQ treated mice did not have significantly different levels of autofluorescence when compared to VC treated mice until 48 h (*p* = <.0001). PQ and TQ groups were also significantly different at both 24 (*p* = <.0001) and 48 h (*p* = <.0001). Crystal violet staining on blood collected at 24 (VC, PQ) and 48 h (TQ) post‐dosing indicated the increased presence of inclusion bodies in both the PQ (24 h) and TQ (48 h) samples, consistent with the formation of Heinz bodies (Figure 3B).

These results suggest that as early as 24 h post drug administration following treatment with PQ and TQ, G6PDd‐huRBCs exhibited increased levels of intracellular ROS, upregulation of PS, as well as elevated levels of autofluorescence, and increased inclusion bodies, both indicative of Heinz bodies.

### 3.3 Evidence of intravascular and extravascular haemolysis in urine post 8‐AQ treatment

Clinical markers of haemolytic toxicity in G6PDd individuals given PQ are bilirubinuria and haemoglobinuria. Urinalysis was performed 50 h post‐dosing with VC, PQ (15 mpk/d × 3), or TQ (5 mpk × 1). Elevated levels of bilirubin, protein and haemolyzed blood were found in the urine of both PQ and TQ treated mice. Non‐haemolyzed blood was elevated in the PQ treated mice only. In addition, urobilinogen was slightly elevated in PQ and TQ treated groups, while glucose levels were largely unaffected in all groups (Table 1).

**TABLE 1:** 8‐AQ treated G6PDd‐huRBC engrafted mice show evidence of intravascular and extravascular haemolysis

	VC			PQ				TQ			Reference Range
	Mu1	Mu2	Mu3	Mu1	Mu2	Mu3	Mu4	Mu1	Mu2	Mu3	
Bilirubin	neg	neg	neg	2+	3+	1+	3+	2+	2+	1+	Neg to 3+
Urobilinogen	norm	norm	norm	norm	0.4	0.2	0.2	norm	0.2	0.2	0.2–1.0 EU/dl
Glucose	neg	neg	neg	neg	1+	1+	neg	neg	neg	neg	Neg to 5+
Protein	neg	neg	neg	2+	4+	2+	3+	2+	2+	3+	Neg to 5+
Hemolyzed blood	neg	neg	neg	4+	4+	3+	3+	2+	2+	4+	Neg to 4+
Non‐Hemolyzed blood	neg	neg	neg	15	10	15	10	neg	neg	neg	5–50 Ery/l
HuRBC% engraftment	67.8	76.8	74.6	76.6	60.6	70.3	69.3	70.1	87.5	79.9	1%–100%

Note

Mice were engrafted with G6PDd‐huRBCs and treated with VC × 3, PQ 15 mpk/d × 3 and TQ 5 mpk × 1 with urine collected 50 h post‐initial doses. Results are for 3 mice per group. Urinalysis results are presented including measurements of bilirubin, urobilinogen, glucose, protein, haemolyzed and non‐haemolyzed blood.

### 3.4 Splenic extramedullary haematopoiesis and CD169+ macrophage depletion in response to 8‐AQ treatment

**FIGURE 4:**
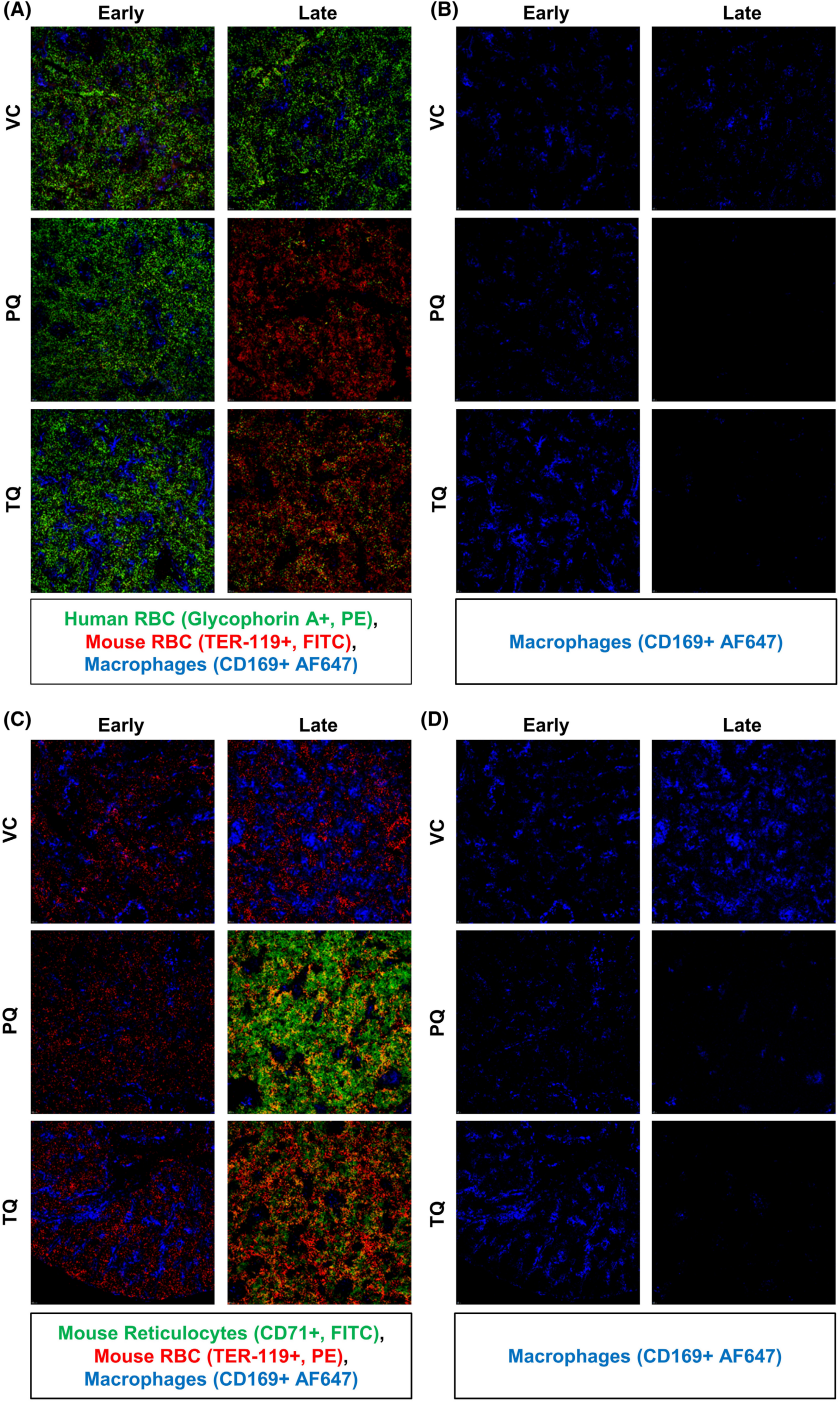
Extramedullary haematopoiesis and splenic CD169+ macrophage populations decrease in response to 8‐AQ treatment. Spleen tissue was collected from G6PDd‐huRBC engrafted mice at 24 h post‐treatment with either VC × 1, PQ 15 mpk × 1 or TQ 10 mpk × 1 and at 5–7 days post‐treatment from mice that received either VC × 3, PQ 15 mpk/d × 3 or TQ 10 mpk × 1. At 24 hour and 5–7 day time points, splenic tissue from all groups was IF stained for huRBCs (Glycophorin‐A+, PE), muRBCS (TER‐119+, FITC) and CD169+ splenic macrophages (CD169+ AF647) (A). At 24 hour and 5–7 day time points, splenic tissue was stained for muRetics (CD71+, FITC), muRBC (TER‐119+, PE) and CD169+ splenic macrophages (CD169+, AF647) (C). CD169+ staining was singularly presented for enhanced visualization of this population at 24 h and 5–7 days for both stained sets (B,D). Representative images were chosen. Images were captured at 10x magnification using an Eclipse TE 2000 microscope (Nikon) and analysed using Slidebook 6.0 software (Intelligent Imaging Innovations)

Increased spleen weight and the rapid rise of muRetics in the peripheral blood in response to PQ and TQ suggested extramedullary haematopoiesis in response to acute anaemia. To evaluate this, spleens were collected from VC, PQ and TQ treated mice post‐dosing at both early (1 day) and late (5–7 days) timepoints and IF stained to identify changes in huRBC (Glycophorin A+), muRBC (Ter119+), and murine erythroblast (CD71+) populations. CD169+ splenic macrophages were evaluated given their critical role in clearance of damaged. IF staining of untreated non‐engrafted and engrafted spleens from NOD/SCID mice revealed limited organization of structural microanatomy consistent with genetic immunodeficiency (Figure S2A). At the early timepoint, equivalent levels of huRBCs were observed in all 3 groups. However, in later post‐treatment timepoints, huRBCs were depleted in PQ and TQ mice (Figure 4A) and corresponded with increased muRBCs (Figure 4C). At the early timepoint, there were no observable differences in levels of murine erythroblasts between groups. While, at the later time point there was evidence of extramedullary haematopoiesis as indicated by increased murine erythroblasts in both PQ and TQ treated mice as compared with VC (Figure 4C). Interestingly, splenic macrophages (CD169+) were present in all groups at the early time point, but were absent in PQ and TQ treated mice by the later observed timepoint (Figure 4B,D).

### 3.5 8‐AQs induced increases in hepatic resident macrophages and iron accumulation

**FIGURE 5:**
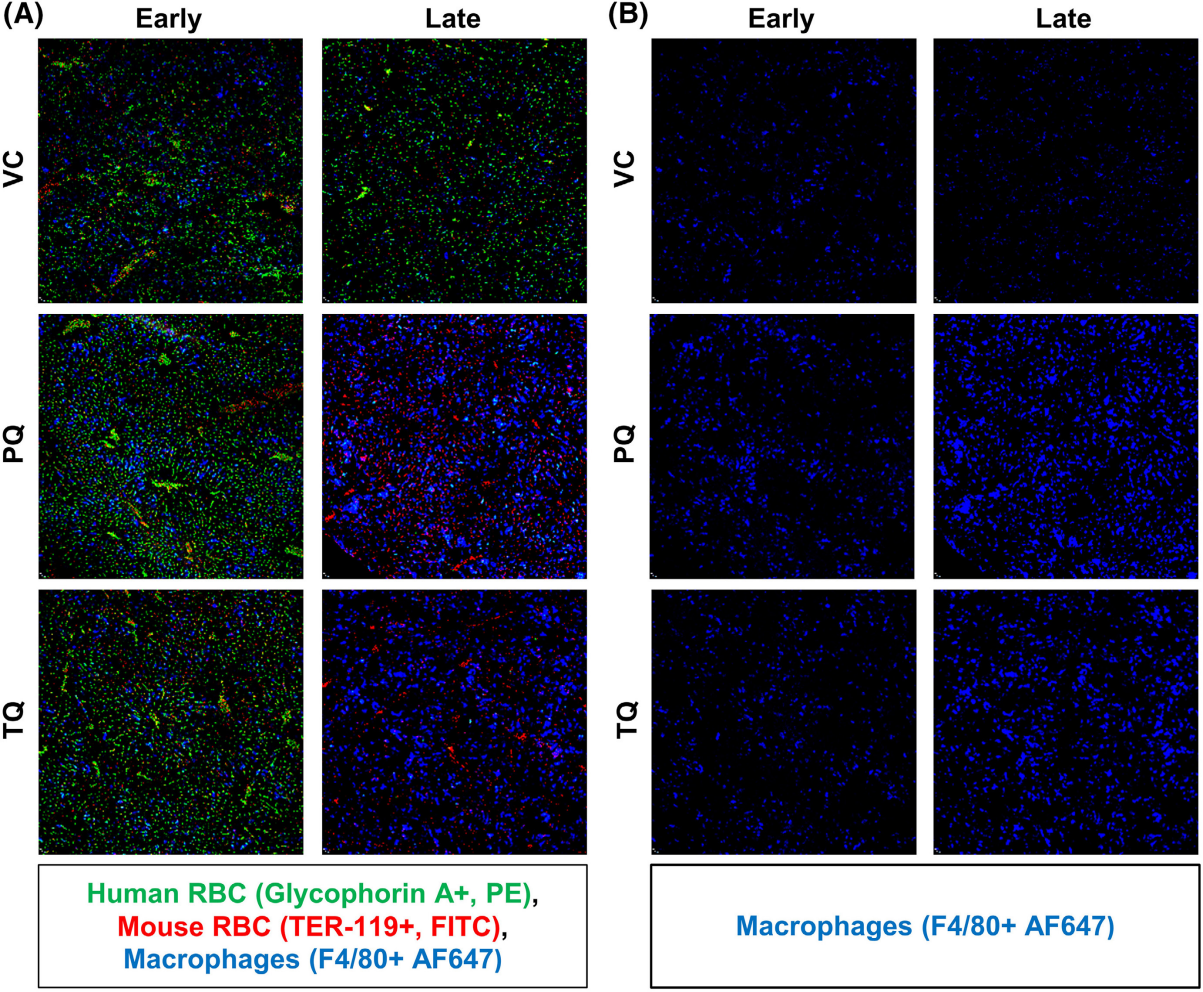
Hepatic macrophage increase in the liver in response to 8‐AQ treatment. Liver tissue was collected at 24 h from G6PDd‐huRBC engrafted mice treated with either VC × 1, PQ 15 mpk × 1 or TQ 10 mpk × 1 and at 5–7 days from mice that received either VC × 3, PQ 15 mpk/d × 3 or TQ 10 mpk × 1. At 24 hour and 5–7 day time points, tissue was stained for huRBC (Glycophorin‐A+, PE), muRBC (TER‐119+, FITC) and F4/80+ macrophages (AF647) (A). Macrophage staining for F4/80 (B) was isolated for better visualization of the population at 24 h and 5–7 days. Representative images were chosen. Images were captured at 10x magnification using an Eclipse TE 2000 microscope (Nikon) and analysed using Slidebook 6.0 software (Intelligent Imaging Innovations)

**FIGURE 6:**
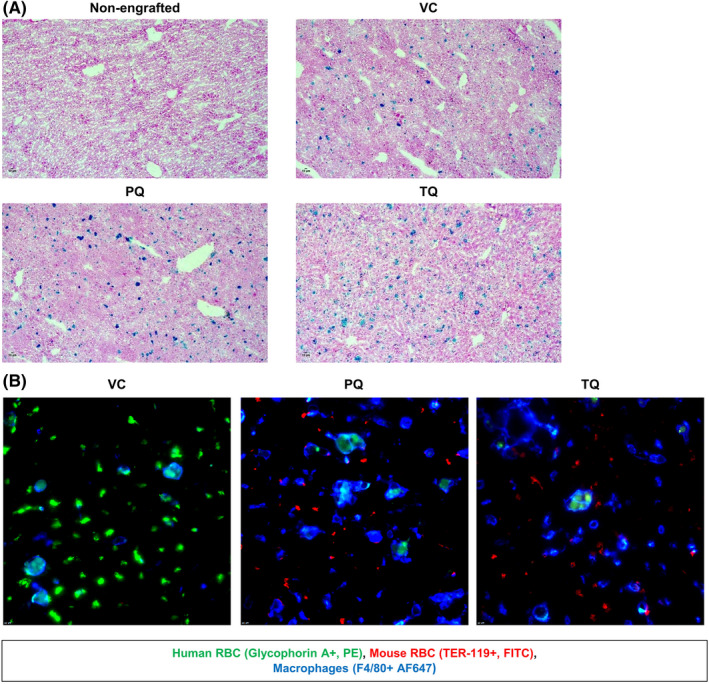
Iron deposits and evidence of hepatic macrophage consumption of G6PDd huRBCs. Prussian blue staining was performed on liver tissue collected from mice on Day 7 that received either: VC × 3, PQ 15mpk/d × 3 or TQ 10 mpk × 1. Nuclear Fast Red staining shows nuclei in red, pink is the background and iron deposits are bright blue in colour. PQ/TQ treated groups were compared with VC and non‐engrafted controls (A). Images were captured at 40x magnification using a Leica DM750 microscope and Leica Acquire software 10.10. Images were further processes using ImageJ software^33^ to add scale bars. Representative images were chosen. At 7 days post‐dosing with VC, PQ or TQ evidence for huRBC engulfment by F4/80+ macrophages were present (B). Liver tissue was stained for huRBCs (Glycophorin‐A+, PE), muRBCS (TER‐119+, FITC) and F4/80+ macrophages (AF647). Representative images were chosen. Image was captured at 60x magnification using an Eclipse TE 2000 microscope (Nikon) and analysed using Slidebook 6.0 software (Intelligent Imaging Innovations)

Both the spleen and liver function to remove damaged RBC.^22^ Therefore, phagocytosis of huRBCs was evaluated in the livers of VC, PQ, and TQ treated mice. Liver tissue was analysed by IF staining for changes to huRBC, muRBC and hepatic macrophage F4/80+ cell populations over time. Results indicated similar huRBC, muRBC and hepatic macrophage F4/80+ levels in the VC group at both early and late timepoints (Figure 5A). The PQ and TQ treated groups showed loss of huRBC populations by the late time point, with increased levels of hepatic F4/80+ macrophages (Figure 5B). These findings indicate that hepatic resident macrophages are responding directly to the 8‐AQ induced haemolysis by recruiting F4/80+ phagocytes to aid in damaged huRBC clearance. In addition, the staining of liver tissue with Prussian Blue to detect ferric iron showed elevated iron deposits indicative of haemosiderosis; the result of increased phagocytosis of RBCs following PQ and TQ treatment (Figure 6A). At the late timepoint, all tissue showed hepatic F4/80+ macrophages engulfing huRBCs (Figure 6B).

## 4 DISCUSSION

The only licensed drugs known to eliminate the hypnozoite reservoir of *P*. *vivax* —PQ and TQ— also cause haemolytic toxicity in G6PDd individuals, severely hampering malaria eradication. Understanding how 8‐AQs cause haemolytic anaemia and how their effects may be mitigated is critical to developing strategies for prevention of haemolytic toxicity. In this study, a well‐characterized humanized mouse model engrafted with G6PDd‐huRBC was used to evaluate and compare events occurring post‐treatment with PQ and TQ and determine the mechanisms of non‐immune haemolytic toxicity. We found that by 24 h post‐treatment with PQ and TQ, G6PDd‐huRBCs showed evidence of damage as indicated by increased levels of intracellular ROS, upregulation of PS and elevated autofluorescence. The observed magnitude and the timing of these changes were different between PQ and TQ, with TQ showing a delayed and lowered response compared to PQ.

Various studies have reported extravascular haemolysis during G6PDd haemolytic anaemia caused by exposure to oxidative agents, with phagocytosis of damaged RBCs in the liver and spleen.^34^, ^35^, ^36^, ^37^, ^38^, ^39^ In contrast, a recent study comparing the haemolytic effects of TQ and PQ on G6PDd volunteers concluded that the effect of TQ was entirely intravascular and PQ primarily extravascular.^15^ Our results suggest that both types were associated with PQ and TQ treatment in G6PDd‐huRBCs. Haemolyzed blood indicative of haemoglobinuria, and urobilinogen was present in the urine of both PQ and TQ treated mice; signifying intravascular haemolysis. While extravascular haemolysis was represented by elevated bilirubin in the urine; resulting from the breakdown of haemoglobin due to RBC removal by phagocytosis in the spleen and liver. These finding are comparable with clinical observations and suggest both intravascular and extravascular haemolysis occur as a result of 8‐AQ treatment.^40^, ^41^, ^42^ Given these findings and conclusions from other studies, we hypothesize that haemolytic events in regard to 8‐AQs and G6PDd‐huRBCs fall on a spectrum of severity and share a common mechanistic route—mediated by the same eryptotic processes—with more oxidatively damaged RBCs succumbing to intravascular haemolysis and less damaged by extravascular removal.^40^

The observed expansion of erythroblasts in the spleen and peripheral blood was indicative of erythropoiesis counterbalancing the loss of huRBCs from haemolysis. When demand for new erythrocytes exceeds the capacity of steady state bone marrow erythrogenesis, the stress erythropoiesis pathway is initiated in the spleen. This pathway is bone morphogenetic protein 4 (BMP4) dependent and is initiated by increased erythrophagocytosis resulting from acute anaemia and inflammatory stress.^24^, ^25^, ^43^, ^44^ In this study, increased erythropoiesis was apparent at 24–48 h post PQ and TQ treatment was observed, suggesting that rapid extramedullary stress erythropoiesis occurred in response to 8‐AQ treatment resulting from increased erythrophagocytosis of oxidatively stressed and eryptotic G6PDd‐huRBCs.

Limited research has been done to look at the effect of oxidative agents on G6PDd RBCs. One published study showed intracellular oxidative stress induced PS membrane scrambling when G6PDd‐huRBC were exposed to the oxidizing agent tert‐butyl‐hydroperoxide.^45^ More common are studies of the effects of oxidizing agents on G6PD‐normal huRBCs. For example, a 2016 study found that eryptosis occurs following treatment of G6PD‐normal huRBCs with TQ.^30^

A recent study found that the haemolytic potential was the same for PQ and TQ in heterozygous females with moderate G6PD activity at clinically relevant dosages.^15^ Our current study suggests that while both compounds had the same end result, loss of G6PDd‐huRBCs, with TQ there was a delay in immediate effects on G6PDd‐huRBCs. This result is consistent the longer half‐life of TQ (~15 days) compared with PQ (~ 3–6 h).^46^ With a single dose of TQ, the delayed effects we observed could allow for a possible approach to minimize toxicity by co‐administration of a protective agent to reduce oxidative damage to G6PD‐RBCs; however, more research is needed. This concept has been explored in vitro with vitamin C, which was found to have protective effects in G6PD‐normal and G6PDd‐RBCs post‐treatment with an oxidizing agent and could serve as a guide for future studies.^47^

PQ and TQ treated mice displayed loss of huRBCs and CD169+ splenic macrophages by 7 days post exposure. These macrophages reside primarily in the metallophilic marginal zone of the spleen and function to recognize and engulf cells expressing surface PS and are likely the population responding to increased levels of PS identified on PQ and TQ treated huRBCs.^1^, ^48^, ^49^ Historical studies performed in humans have shown that radioactively labelled RBCs following exposure to PQ primarily localized to the spleen.^37^, ^50^ In another study in rats, the same technique was utilized with 5‐hydroxyprimaquine, and concluded splenic macrophages were responsible for clearance of treated RBCs.^38^ We hypothesize that increased phagocytosis of RBCs displaying PS in spleens of 8‐AQ treated mice led to the observed loss of the CD169+ macrophage population via iron cytotoxicity induced ferroptosis. Other studies have also reported that elevated erythrophagocytosis and the release of intracellular haeme can lead to macrophage loss.^51^, ^52^ Similar evidence for ferroptosis was derived using splenic red‐pulp macrophages when storage‐damaged RBCs were transfused into mice.^53^

Elevated hepatic macrophages were observed in PQ and TQ treated G6PDd‐huRBC mice, along with evidence of haemosiderosis, consistent with clinical reports of iron overload in various haematological disorders.^54^ The liver has been previously shown to function as the primary site of erythrophagocytosis under abnormal conditions in a mouse model.^52^ A recent publication by Liu et al.,^55^ provided evidence that extracellular heme released during intravascular haemolysis increases hepatic macrophage levels and erythrophagocytosis, helping to mitigate haemolytic injury. Concurrent intravascular and extravascular haemolysis in our PQ and TQ treated mice dictate that elevated levels of haemoglobin and heme are present and likely driving the same macrophage recruitment to the liver where increased levels of hepatic F4/80 macrophages were observed.

In summary, the use of the humanized G6PDd‐huRBC mouse model allowed us to follow effects of 8‐AQ induced haemolysis in a relevant in vivo system. These studies suggest that G6PDd‐huRBCs have early and differing physiological responses to PQ and TQ, including increased ROS, markers for eryptosis and oxidative stress. Damaged RBCs subsequently succumb to intravascular haemolysis or are removed extravascularly by phagocytosis in the spleen and liver. Excessive phagocytosis of PQ‐ and TQ‐treated huRBCs triggers ferroptosis in splenic macrophages, and the spleen begins functioning as the primary site for extramedullary haematopoiesis as part of the stress erythropoiesis response. As splenic macrophages are lost, increases in hepatic macrophage populations are observed, resulting in huRBC clearance. The findings presented here are an important contribution to explaining the physiological responses and mechanisms of haemolytic toxicity in G6PDd individuals.

## AUTHOR CONTRIBUTION

**Siobhan Flaherty:** Conceptualization (lead); Data curation (lead); Formal analysis (lead); Investigation (lead); Methodology (lead); Project administration (lead); Resources (supporting); Validation (lead); Visualization (lead); Writing – original draft (lead); Writing – review & editing (lead). **Pamela Strauch:** Conceptualization (equal); Data curation (equal); Investigation (equal); Methodology (equal); Project administration (equal); Resources (equal); Validation (equal); Visualization (equal); Writing – review & editing (equal). **Mahdi Maktabi:** Data curation (supporting); Formal analysis (supporting); Investigation (supporting); Project administration (supporting). **Brandon S. Pybus:** Conceptualization (supporting); Writing – review & editing (supporting). **Gregory Reichard:** Conceptualization (supporting); Writing – review & editing (supporting). **Larry Walker:** Conceptualization (supporting); Funding acquisition (lead); Writing – review & editing (supporting). **Rosemary Rochford:** Conceptualization (lead); Data curation (equal); Formal analysis (equal); Funding acquisition (lead); Investigation (equal); Methodology (equal); Project administration (equal); Resources (lead); Supervision (lead); Validation (lead); Visualization (lead); Writing – original draft (equal); Writing – review & editing (equal).

## CONFLICT OF INTEREST

The authors declare that the research was conducted in the absence of any commercial or financial relationships that could be construed as a potential conflict of interest.

## Supporting information

Fig S1‐S2

Supplementary Material

## Data Availability

The data that support the findings of this study are available from the corresponding author upon reasonable request. For original data, please contact siobhan.flaherty@cuanschutz.edu.
