# Supplementary figures and images for: Mechanisms of 8‐aminoquinoline induced haemolytic toxicity in a G6PDd humanized mouse model
Source: J Cell Mol Med. 2022 Jun 3;26(13):3675–86. doi: 10.1111/jcmm.17362 (PMC9258708; doi:10.1111/jcmm.17362)

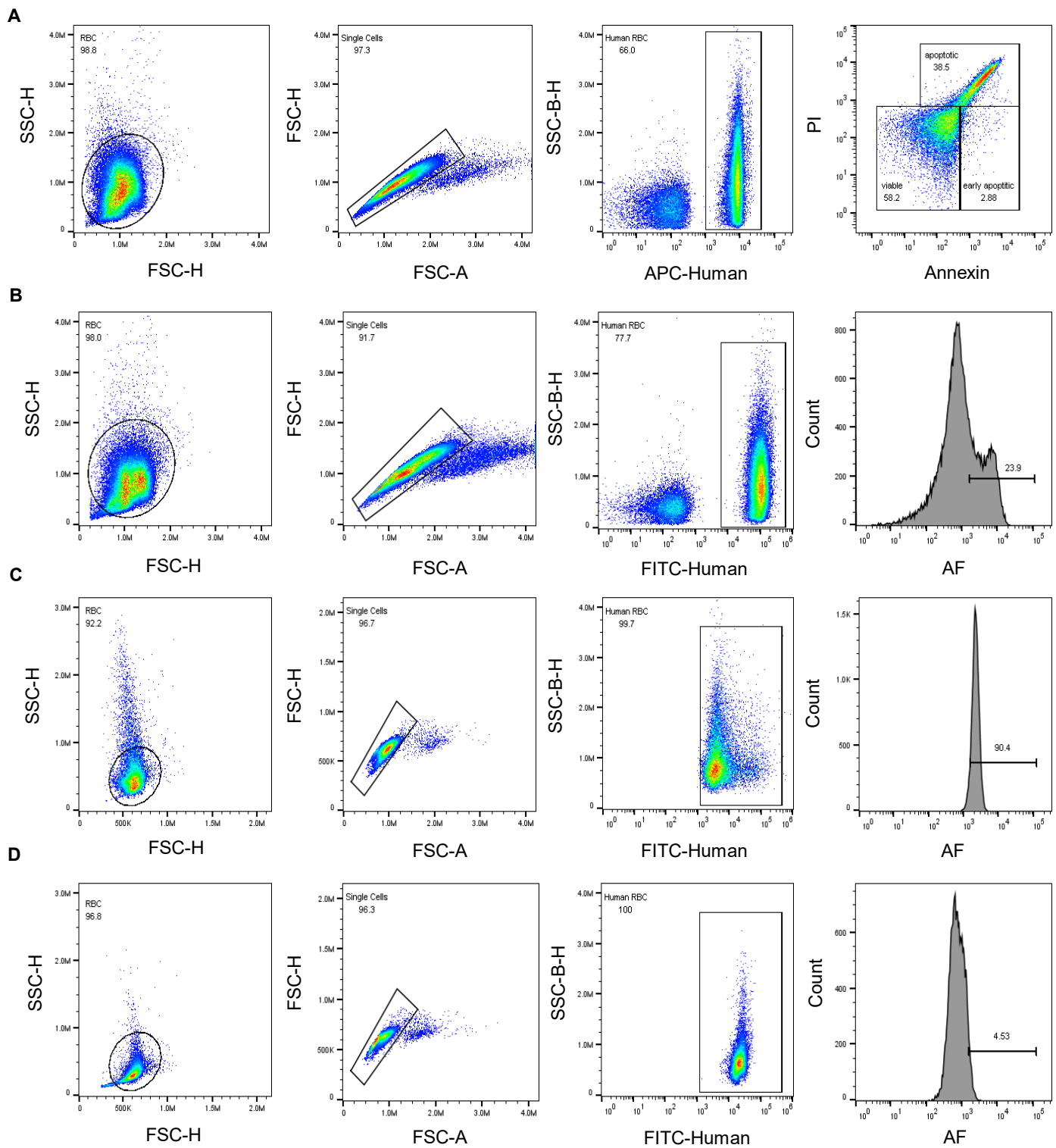

Supplemental Figure 1

A

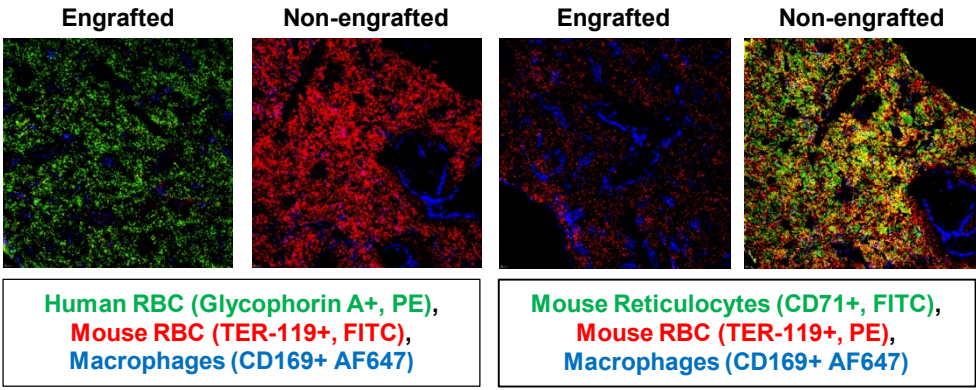

B

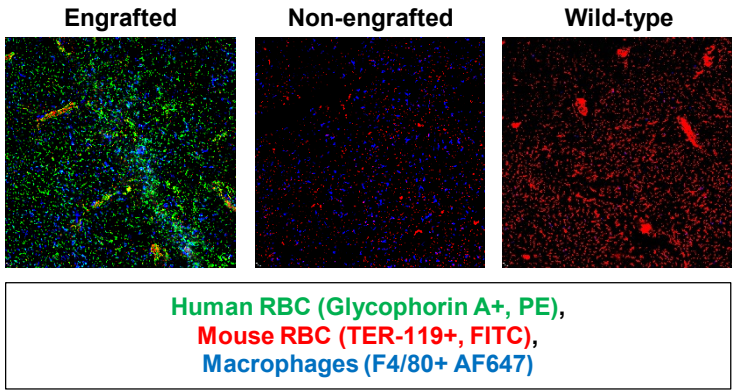

Supplemental Figure 2

Supplement: Supplementary file 1 — Fig S1‐S2 [file JCMM-26-3675-s001.pdf]
