# Supplementary material for: Mechanisms of 8‐aminoquinoline induced haemolytic toxicity in a G6PDd humanized mouse model
Source: J Cell Mol Med. 2022 Jun 3;26(13):3675–86. doi: 10.1111/jcmm.17362 (PMC9258708; doi:10.1111/jcmm.17362)
Supplement: Supplementary file 2 — Supplementary Material [file JCMM-26-3675-s002.docx]

**Supplemental Figure 1. Gating strategy to determine Annexin/PI and autofluorescence changes in response to treatment.** Mice were engrafted with G6PDd-huRBCs and treated with VC x 1, PQ 15 mpk x 1 or TQ 10 mpk x 1. Flow cytometry experiments included measuring changes to Annexin/PI using the presented gating strategy (A). Gating for changes in autofluorescence was defined through the use of the positive control—phenylhydrazine—(C) and negative control—untreated G6PDd-huRBCs—(D) with gates applied to huRBCs from experimental groups (B).

**Supplemental Figure 2. Immunofluorescent spleen and liver tissue control staining.** Spleen tissue was collected from engrafted and non-engrafted G6PDd-huRBC untreated mice and stained for huRBCs (Glycophorin-A+), muRBCS (TER-119+), muRetics (CD71+) and splenic macrophages (CD169+) (A). **In** addition, untreated engrafted and non-engrafted hepatic tissue and wild type C57-BL/6J mouse hepatic tissue was stained for huRBCs (Glycophorin-A+), muRBCS (TER-119+) and macrophages (F4/80+) (B). Images were captured at 10x magnification using an Eclipse TE 2000 microscope (Nikon) with NIS Elements version 4.2 software and viewed with NIS Elements Viewer version 4.11.0 software (Nikon).
